# Evaluation of long-term outcomes with intrathecal opioid treatment: a comparison utilizing data derived from pain clinic populations in Australia and New Zealand

**DOI:** 10.3389/fpain.2025.1527371

**Published:** 2025-02-14

**Authors:** Elouise Rose Comber, Jenny Strong, Orla Moore, Asaduzzaman Khan, James O’Callaghan, Benjamin Manion, Brendan Joseph Moore, Maree Therese Smith

**Affiliations:** ^1^ Faculty of Science, School of Chemistry and Molecular Biosciences, The University of Queensland, Brisbane, QLD, Australia; ^2^ Faculty of Health and Behavioural Sciences, School of Health and Rehabilitation Sciences, The University of Queensland, Brisbane, QLD, Australia; ^3^ Faculty of Medicine, School of Medicine, The University of Queensland, Brisbane, QLD, Australia; ^4^ Axxon Pain Medicine, Brisbane, QLD, Australia; ^5^ Faculty of Medicine, School of Biomedical Sciences, The University of Queensland, Brisbane, QLD, Australia

**Keywords:** intrathecal (IT), opioid, electronic persistent pain outcomes collaboration (ePPOC), psychosocial outcomes, longterm IT opioid, chronic non-cancer pain

## Abstract

**Introduction:**

An obstacle to analysis of the long-term effectiveness of intrathecal (IT) opioids is absence of historical patient baseline data. The electronic Persistent Pain Outcomes Collaboration (ePPOC) is an initiative of the Faculty of Pain Medicine of the Australian and New Zealand College of Anaesthetists. Recently published ePPOC data has provided justifiable surrogate baseline data allowing opportunities for pain outcomes research into select patient treatment groups. Our aim was to compare long-term outcomes of IT opioid therapy with a surrogate baseline utilizing a large ePPOC data set for patients at the time of initial presentation to 36 pain clinics in Australia and New Zealand.

**Methods:**

Study participants were 49 consenting patients receiving IT opioids as part of a long-term pain management regime for treating chronic non-cancer pain. Their data were compared with the large ePPOC data set (*n* = 13,343). The questionnaires comprised a demographic questionnaire, the Brief Pain Inventory, the Depression, Anxiety and Stress Scale, the Pain Catastrophizing Questionnaire, and the Pain Self-Efficacy Questionnaire.

**Results:**

Compared with the ePOCC group, participants who received IT opioids long-term for the relief of chronic non-cancer pain reported significantly lower (*p* ≤ 0.001) pain severity (4.3 vs. 6.4), and pain interference scores (5.5 vs. 7), significantly lower depression (20.2 vs. 13.7), anxiety (9.6 vs. 14.1), stress (15.5 vs. 21), rumination (6.9 vs. 10), magnification (3.8 vs. 5.9), helplessness (9.7 vs. 14.1), general catastrophizing (20.4 vs. 29.8), and higher self-efficacy (29.5 vs. 20.7).

**Discussion:**

The observed improvements in all measured pain variables have occurred in the context of comprehensive pain management, and therefore, may be attributable to pain reduction and not directly to IT opioid use or the device itself. Favourable pain management outcomes, in a select patient treatment group utilizing long-term IT opioid therapy, were demonstrated using the large-data ePPOC initiative, highlighting the research opportunities it provides.

## 1 Introduction

The electronic Persistent Pain Outcomes Collaboration (ePPOC) was established in 2013 as an initiative of the Faculty of Pain Medicine of the Australian and New Zealand College of Anaesthetists. This initiative has grown to include data from more than 100 adult and paediatric services across Australia and New Zealand. Since its implementation, ePPOC has enabled standardized routine collection of data from pain clinics across Australia and New Zealand (1, 2). The ePPOC data set provides a valuable data pool for pain research, with data collected from 80 pain services and over 20,000 individuals over almost 10 years (1). The ePPOC questionnaire consists of well-established, internationally recognized pain assessment scales aligned with the biopsychosocial model of pain.

Nicholas et al. published baseline data from ePPOC for patients (*n* = 13,343) at time of initial presentation to 36 pain clinics across Australia and New Zealand for pain management (3). This population had a mean age of 52.7 years, 58.4% were female, 86.7% were non-indigenous, 69.5% were born in Australia, 98.7% had chronic pain due to non-cancer causes, and 33.3% of participants were unemployed due to pain. For 46.8% of individuals, the duration of their pain was more than 5 years, for 21.4% of participants the triggering event was an injury at work or school, and the back/spine/sacrum was the main pain site for 47.9% of participants.

These data provided a justifiable surrogate baseline for comparison to assess effectiveness of treatments in select patient groups. Further publications emphasize the importance and versatility of ePPOC data as a comparison to assess the effectiveness of treatment in individual pain management centres or in selected subgroups of patients (3, 4).

In this study we utilized the ePPOC data, as intended, to compare the effectiveness of pain management over time in a specific group of long-term chronic non-cancer pain sufferers who have implanted intrathecal (IT) drug delivery systems (IDDS) to administer IT opioids, as part of a comprehensive approach to their pain management (4). Although the ePPOC dataset has a large sample size and uses standardized measures which provide invaluable baseline data, it is important to acknowledge that there may be demographic and clinical differences in the dataset in the current study compared to ePPOC. The ePPOC data allowed comparison of current patient data to a surrogate baseline in the absence of actual historical baseline data for this group. The use of questionnaires which are incorporated in ePPOC, allows standardized, non-invasive quantification of the impact of IT opioids on patients' pain intensity, quality of life and daily functioning.

IDDS are surgically implanted and comprise an intrathecal catheter attached to a subcutaneous, programmable, refillable pump, delivering continuous IT infusion (5). Although the process of IT pump implantation is invasive, the IT route of drug administration may be utilized to achieve enhanced pain relief when traditional routes of administration prove inadequate, due to poor efficacy and/or dose-limiting adverse effects (6).

In line with published international best practice, most patients receiving long term IT medications for pain relief, receive polyanalgesia including some off-label medication use (7). IT adjuvant agents added to IT opioids in the IDDS include clonidine and local anesthetics with baclofen included to treat chronic pain and spasticity (8). This enables a small fraction of the typical systemic analgesic drug doses to be delivered close to spinal cord receptors/ion channels that mediate pain relief, potentially exceeding the efficacy of oral dosing regimens whilst minimizing side effects due to metabolically-derived neuroexcitatory metabolites of the opioid analgesics, morphine and hydromorphone (9). This increases analgesic effects and duration, limits the need to consistently increase dosage, decreases systemic side effects, reduces systemic exposure to analgesic/adjuvant medications and prevents missed dosage or overdosage (10, 11). However, concordant with other dosing routes, analgesic tolerance may still develop and there remains potential for addiction liability (12, 13). Consistent with any surgically inserted medical device there are potential complications related to the implant procedure (14). Other complications include technical IDDS malfunction, IT catheter fracture or migration interrupting IT drug delivery, infection, granuloma formation, particularly at the catheter tip, as well as refilling and programming errors leading to over or under dosing (14).

Nadeau and colleagues (14) noted that in the United States, chronic pain is often undertreated due to preconceptions of health professionals around imminent opioid addiction, often due to limited medical training in chronic pain management. This includes a lack of awareness of the approximately 13-fold variability in opioid dosing, dependent largely on genetic variability in opioid metabolism (14). Side effects of opioids are often concerns for clinicians but these may be eliminated by swapping to alternative opioids (14). Few clinicians are aware that fully adequate treatment of depression can also yield large gains in pain control (15).

Recent studies have reached varied conclusions regarding the efficacy of IT opioids, delivered via implanted pumps, for chronic pain management. Decreases in pain scores were reported in most studies (10, 16–21), but some studies found no change (3, 22). There remains no clear picture of the impact of IT opioids on the biopsychosocial outcomes of these patients and therefore, this study aimed to address this knowledge gap.

This research study was designed to use existing validated, patient-reported outcome measures, utilized in ePPOC to measure pain intensity and pain interference, depression, anxiety and stress, pain catastrophizing and self-efficacy (23).

Our aim was to compare long-term outcomes of IT opioid therapy in patients with chronic non-cancer pain with a surrogate baseline utilizing a large ePPOC data set for patients at the time of initial presentation to 36 pain clinics in Australia and New Zealand. This research had two objectives: firstly, to use questionnaires consistent with ePPOC to document and describe characteristics of a cohort of patients receiving IT opioids via an implanted pump, managed by doctors at a private pain management practice and perform a stratified analysis of this cohort of patients based on demographic characteristics; and secondly, to compare the current patient cohort receiving long term IT opioid delivery with corresponding ePPOC data from a large cohort of patients, at initial presentation to a pain clinic, not receiving intrathecal opioids, who were seeking management for chronic pain across 36 Australian and New Zealand pain clinics. We hypothesized that in the cohort of individuals studied who had chronic non-cancer pain and who were receiving longterm IT opioid/adjuvant analgesic therapy, the IT route of analgesic/adjuvant agent delivery has contributed to positively improved outcomes.

## 2 Methods

### 2.1 Study design

A cross-sectional study design was used for data collection with patients experiencing chronic non-cancer pain and receiving opioid analgesics via chronically implanted IT pumps. Ethical clearance was granted from The University of Queensland Human Research Ethics Committee (Project ID 2022/HE000145), Brisbane Private Hospital and Greenslopes Private Hospital. This study did not focus on individualized data; rather, a detailed cohort description was necessary for comparison to previously published data (3). Treating pain specialists determined participant eligibility via a review of medical charts of this special patient population, the majority of whom were legacy patients whose original pain medicine specialist had retired. All patients receiving intrathecal opioids via an implanted pump who were being treated at the practice (*n* = 95) were assessed for eligibility. If eligible, patients were invited by their treating doctor to participate, and if interested, were given or posted a “Participant Information Sheet” and a “Participant Consent Form”. Where necessary, patients were also given a self-addressed envelope for returning signed consent forms. The number of participants who declined to participate in the study was recorded. Data were collected by semi-structured questionnaire interviews with participants. Questionnaire interviews were completed in person at Brisbane Private Hospital or Greenslopes Private Hospital, or by telephone. Two questionnaires were completed by the participant via hardcopy questionnaire and a further three participants had partners assist with answers. Questionnaire completion took approximately 45 min. To limit variation in data collection, only two researchers administered the questionnaires and were trained in interview techniques by an experienced member of the research team (JS). In semi-structured interviews, a standardized script was used for the introduction and questions were read word-for-word by interviewers. Following questionnaire completion, participant involvement in the study was complete and responses were anonymized and allocated a number. The IT opioid-managed cohort data set was then compared with the recently published ePPOC data set of 13,343 patients with chronic pain at the time of initial presentation for treatment to 36 pain clinics across Australia and New Zealand (3). A useful comparison is gained by comparing data from the current cohort, for which there were no historical baseline data before pump insertion, to the larger ePPOC data set (3), acting as a substitute baseline. No power calculations were performed to determine the sample size of patients with IT opioid pumps for the treatment of chronic pain, as this study used a purposive sample.

### 2.2 Participants

Patients (*n* = 49) with chronically implanted pumps for IT opioid delivery (with and without analgesic adjuvant agents) for the pharmacological treatment of chronic non-cancer pain were recruited. Inclusion criteria were: (a) 18 years of age or older, (b) diagnosed with chronic non-cancer pain, (c) have an implanted IT pump (Medtronic) delivering chronic opioid therapy, and (d) be competent in English to provide written informed consent and complete questionnaires during the semi-structured interview. Patients were excluded from the study if: (a) they had a major psychiatric disorder such as psychosis or schizophrenia or (b) they had a significant cognitive impairment that would prevent questionnaire completion. Minority groups such as culturally and linguistically diverse people and Aboriginal and Torres Strait Islander communities were not excluded from this study.

### 2.3 Measures

This study used pain questionnaires identical to those used in ePPOC. The questionnaire subset derived from ePPOC comprised a set of patient-reported outcome measures including the Brief Pain Inventory (BPI), 21-item Depression, Anxiety, and Stress Scale (DASS), Pain Self-Efficacy Questionnaire (PSEQ) and the Pain Catastrophizing Scale (PCS) (24). The demographic questionnaire included questions from ePPOC, with the addition of questions specifically related to intrathecal pump use. This was used to gather a clear general participant description.

The BPI, developed by Cleeland et al. (25), has two subscales: Pain Severity and Pain Interference. The Pain Severity Questionnaire measures pain intensity over 24 h on an 11-point scale (0 = “no pain” to 10 = “pain as bad as you can imagine”), based on four questions. The Pain Interference Questionnaire asks a patient to rate how pain has interfered with seven behaviors in the past 24 h on an 11-point scale (0 = “does not interfere” to 10 = “completely interferes”). The BPI has high reliability and validity (Cronbach's alpha = 0.84; Pearson's correlation coefficient = 0.81) (18, 19).

The DASS-21 measures symptoms of depression, anxiety and stress that a person has experienced over the past week (26). The subscales can be considered separately or grouped to form a general distress construct (27). In this study, a general distress score was not calculated. The DASS-21 uses 21 questions answered on a four-point scale (0 = “did not apply to me at all” to 3 = “applied very much, or most of the time”). Questions enquire about experiences such as agitation, ability to relax, physical manifestations (of depression, stress and anxiety) and emotions (26). Sinclair et al. (11) reported high reliability (Cronbach's alphas = 0.91, 0.80 and 0.84 for depression, anxiety and stress subscales respectively). Good validity was indicated by correlation coefficients with the Beck Anxiety Inventory of 0.81 and 0.74 with the Beck Depression Inventory for anxiety and depression respectively (28). Good validity was also indicated with a correlation coefficient of 0.64 with the Perceived Stress Scale (29).

The PCS measures the thoughts and feelings a patient has related to their pain, using 13 questions rated on a five-point scale (0 = “not at all” to 4 = “all the time”) (20). The questionnaire covers three subscales: rumination, magnification and helplessness (20). The PCS has high reliability (Cronbach's alpha = 0.90) (30), and validity (r = −0.54 with the PSEQ) (1).

The PSEQ, developed by Nicholas et al. (31), asks 10 questions to determine a patient's confidence to perform certain activities despite their pain. Answers are given on a seven-point scale, with higher scores indicating higher self-efficacy (0 = “not at all confident”, to 6 = “completely confident”) (31). The PSEQ has high reliability and validity (Cronbach's alpha = 0.94; r = −0.54 with PCS) (1, 6).

### 2.4 Statistical analysis

Analyses were performed using the Statistical Package for the Social Sciences (SPSS) (version 28). Proportions and “n” values were calculated for categorical variables and means, standard deviations, medians and interquartile ranges were calculated for continuous variables. Correlations were also calculated using SPSS. Comparison with the ePPOC data set was performed using t-tests via online statistical calculators for continuous variables when comparing group means (21). Categorical data were analyzed using an online statistical calculator to perform chi-square tests where appropriate (32). The significance level was set at *p* < 0.05 for all statistical tests. Data were presented for overall comparison between the ePPOC sample and the current study data.

## 3 Results

### 3.1 Demographic details and pain-related characteristics

Of the 52 patients approached during recruitment in the period May to September 2022, 49 were recruited and three declined participation due to personal or logistical reasons. This study did not omit any participants based on exclusion criteria. Demographic characteristics, pain-related characteristics and IT pump-related characteristics of the recruited cohort are displayed in Tables 1–3 respectively. Table 1 shows the number of participants with IT pumps per characteristic (n) and the percentage frequency (%) for each demographic characteristic. When compared with the ePPOC data participants, patients receiving chronic IT opioid therapy via a chronically implanted pump were more likely to work full time or be retired, but less likely to work part time or be unemployed due to pain. For greater insight, Supplementary Results Table S1 contains the descriptive comments provided by study participants during the interviews.

**Table 1:** Demographic characteristics.

	Nicholas et al. ePPOC dataset from (3) (*n* = 13,250)		Current study (*n* = 49)		
	*n*	%	*n*	%	
Age groups (years)					
≤50	6,003	45.3	6	12.2	***
51–60	3,247	24.5	12	24.5	
61–70	2,067	15.6	19	38.8	
71–80	1,346	10.2	12	24.5	
>80	587	4.4	0	0.0	
Mean age (SD)	52.7 (15.76)		63.1 (9.5)		
Sex					
Female	7,794	58.4	28	57.1	
Male	5,545	41.6	21	42.9	
Indigenous status					
Non Indigenous	11,556	86.7	49	100	
Aboriginal	526	3.9	0	0.0	
Torres Strait Islander	34	0.3	0	0.0	
Maori	56	0.4	0	0.0	
Country of birth					
Australia	8,998	69.5	41	83.7	
New Zealand	330	2.5	1	2.0	
Other	3,614	27.9	7	14.3	
Department of Veteran Affairs patient					
Yes	–	–	10	20.4	
No	–	–	39	79.6	
Served in the Australian Defence Force					
Yes	–	–	11	22.4	
No	–	–	38	77.6	
Current work status					
Full time	1,237	9.3	6	12.2	
Part time	1,016	7.6	2	4.1	
Retired	3,245	24.4	24	49.0	
Unemployed due to pain	4,442	33.3	14	28.6	
Unemployed not due to pain	704	5.3	0	0.0	
Home duties	1,726	13	0	0.0	
On leave due to pain	781	5.9	0	0.0	
Studying	508	3.8	0	0.0	
Retraining	112	0.8	0	0.0	
Limited hours	544	4.1	0	0.0	
Voluntary work	382	2.9	1	2.0	
Other	–	–	2	4.1	

Where appropriate, categorical variables were analysed using chi-square tests; * = *p* < 0.05, ** = *p* < 0.01, *** = *p* < 0.001, ns = no significance.

**Table 2:** Pain-related characteristics.

	Nicholas et al. ePPOC dataset from (3)		Current study (*n* = 49)		
	*n*	%	*n*	%	
Pain duration					
<3 months	321	2.6	0	0.0	
3–12 months	1,587	12.7	0	0.0	
12 months—2 years	1,838	14.7	0	0.0	
2 years—5 years	2,916	23.3	2	4.1	
>5 years	5,872	46.8	47	95.9	
Triggering event					
Injury at home	903	7.1	4	8.2	
Injury at work/school	2,729	21.4	15	30.6	
Injury in another setting	955	7.5	7	14.3	
Motor vehicle accident	1,213	9.5	3	6.1	
Related to cancer	222	1.7	0	0.0	
Medical condition other than cancer	1,404	11	5	10.2	
After surgery	1,410	11.1	5	10.2	
No obvious cause	2,296	18	10	20.4	
Other	1,621	12.7	–	–	
Site with the most pain					
Head/face	739	7	0	0.0	
Neck	812	7.7	2	4.1	
Shoulder/upper limbs	1,567	14.8	0	0.0	
Back/spine/sacrum	5,073	47.9	32	65.3	
Lower limbs	1,409	13.3	5	10.2	
Whole body	122	1.2	7	14.3	
Abdomen/hip	865	8.2	3	6.1	
Number of pain sites					
Not stated	402	3	–	–	
1	3,097	23.2	12	24.5	
2	3,790	28.4	13	26.5	
3	2,752	20.6	13	26.5	
4	1,931	14.5	3	6.1	
5	932	7	2	4.1	
6	439	3.3	6	12.2	
Compensation case					
Yes	1,699	13.4	16	32.7	
No	11,008	86.6	33	67.3	
Work hours affected by pain					
No	10,171	84.4	3	6.1	
Yes	1,882	15.6	43	87.8	
Not applicable	–	–	3	6.1	
Work type affected by pain					
No	10,564	87.7	19	38.8	
Yes	1,487	12.3	7	14.3	
Not applicable			23	46.9	
Diagnosed health conditions^▴^					
Respiratory	–	–	13	26.5	
Heart	–	–	9	18.4	
Cancer	–	–	3	6.1	
Diabetes	–	–	5	10.2	
Anxiety	–	–	6	12.2	
Depression	–	–	14	28.6	
Other	–	–	34	69.4	
Number of analgesic medications (excluding IT medications)					
0	593	6.0	21	42.9	***
1	1,681	17.0	21	42.9	
2	2,506	25.3	5	10.2	
3	2,409	24.3	0	0.0	
≥4	2,719	27.4	2	4.1	
Number of other oral medications					
0	–	–	4	8.2	
1	–	–	12	24.5	
2	–	–	4	8.2	
3	–	–	4	8.2	
≥4	–	–	25	51.0	

^▴^ Some participants had more than one diagnosed health condition. Where appropriate, categorical variables were analysed using chi-square tests; * = *p* < 0.05, ** = *p* < 0.01, *** = *p* < 0.001, ns, no significance.

**Table 3:** It pump-related characteristics for the current study (*n* = 49).

	*n*	%
Approximate duration of IT pump use		
<5 years	7	14.3
5–10 years	1	2.0
11–15 years	7	14.3
16–20 years	13	26.5
>20 years	21	42.9
Medications contained in the IT pump		
Opioid	22	44.9
Opioid + anaesthetic	12	24.5
Opioid + alpha-adrenergic agonist	7	14.3
Opioid + anaesthetic + alpha-adrenergic agonist	7	14.3
Opioid + skeletal muscle relaxant	1	2.0

Table 2 below displays the number of participants (*n*) and the percentage frequency (%) for pain-related characteristics. Most participants (95.9%) had lived with chronic pain for more than five years. The leading triggering event was an injury at work (30.6%), with the second highest causal event identified as “no obvious cause” (20.4%). Further discussion with participants who stated “no obvious cause” for their pain often revealed the occurrence of accidents several years prior to pain onset. The back/spine/sacrum was the most common pain site (65.3%) and was more frequently reported as lower back pain.

Primary opioids (dose range; mean) used in the study group were as follows: morphine (0.5–17 mg/day; 3 mg/day), hydromorphone (0.15–2.85 mg/day; 1.2 mg/day), fentanyl (7–24 µg/day; 12.26 µg/day) and sufentanil (6.2–13.59 µg/day; 8.76 µg/day). Adjuvant agents (dose range; mean) were as follows: clonidine (30–266.5 µg/day; 116.43 µg/day), ropivacaine (0.23–28.2 mg/day; 2.95 mg/day), bupivacaine (0.54–1 mg/day; 0.75 mg/day), and baclofen (0.4–1.4 mg/day; 0.9 mg/day).

Participants in this sample presented with co-morbidities including respiratory conditions (26.5%), heart conditions (18.4%), cancer (6.1%), diabetes (10.2%), anxiety (12.2%), depression (28.6%) and other health conditions (69.4%). 42.9% of participants were taking no oral analgesic medications and 42.9% were taking only one oral analgesic regularly. This contrasted with Nicholas et al.'s (3) study, where 27.4% of study participants were taking four or more oral analgesics. The difference between the number of oral opioids consumed between the two studies is statistically significant (*p* < 0.001). In the current study, 51% of participants were taking a total of four or more other medications, unrelated to their pain. Analgesic adjuvants were not included in these totals and medications were only included if they were taken three or more times per week. Vitamins, supplements and topical ointments were not recorded.

Table 3 displays the number of participants (n) and the percentage frequency (%) for chronically implanted pump characteristics for delivery of IT opioids (with or without adjuvant agents). In the current study sample, 42.9% of participants had their pump *in situ* for more than 20 years and 69.4% of patients had their pump *in situ* for more than 16 years. However, 13 patients were unsure of the exact year of implantation; legacy patient medical files did not always date back to the time of pump insertion. This was due to the pump implantation procedure being performed by pain specialists external to the private pain practice team, with subsequent transfer of care to the current pain medicine practice. Participants in the current study were administered IT opioids mixed with a saline solution, with or without adjuvant agents. The addition of adjuvant agents to opioids, administered via the intrathecal pump, was routinely considered in the course of clinical management of these patients to optimize pain control and minimize the required opioid dose. Supplementary Table S2 details specific IT pump medications.

**Figure 1:**
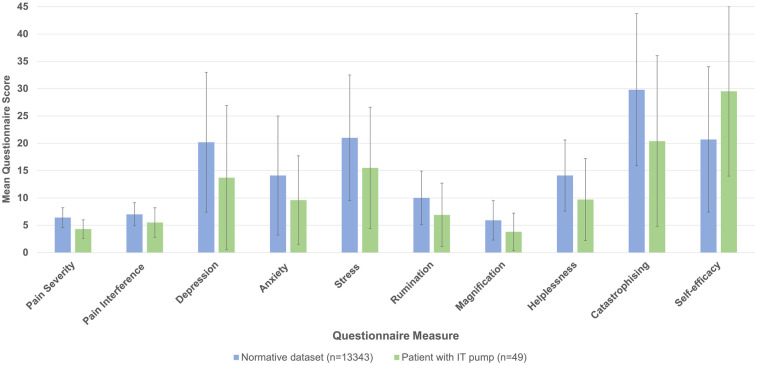
Mean BPI, DASS, PCS and PSEQ outcomes for the current study compared with Nicholas et al.'s (3) study. Standard deviations are shown by error bars.

Figure 1 displays the mean questionnaire scores for the subscales and total scores of the BPI, DASS, PCS and PSEQ for both the current study and Nicholas et al.'s (3) study. Tabular data for this comparison can be found in Supplementary Table S3. Standard deviations are shown by error bars. Participants with chronically implanted pumps for delivery of IT opioids reported significantly lower pain severity (4.3 vs. 6.4; *p* < 0.001) and pain interference scores [5.5 [moderate] vs. 7 [severe]; *p* < 0.001]. The current sample also showed significantly lower scores for depression [20.2 vs. 13.7 (both moderate); *p* < 0.001], anxiety [9.6 vs. 14.1 (both moderate); *p* < 0.01], stress [15.5 [mild] vs. 21 [moderate]; *p* < 0.001], rumination (6.9 vs. 10; *p* < 0.001), magnification (3.8 vs. 5.9; *p* < 0.001), helplessness (9.7 vs. 14.1; *p* < 0.001) and general catastrophizing (20.4 vs. 29.8; *p* < 0.001). Nicholas et al.'s (3) study reported a mean pain catastrophizing score only slightly below the PCS cut off for clinical relevance of 30 (20). However, participants in the current study with chronically implanted pumps delivering IT opioids had scores substantially below this cut-off limit. Significantly higher self-efficacy was observed for individuals receiving IT opioids (29.5 vs. 20.7; *p* < 0.001).

## 4 Discussion

There is increasing awareness of the need for comprehensive evaluation of the chronic pain experience (33). In the context of patients with chronic non-cancer pain receiving longterm IT opioid delivery for pain management, this delivery route has been used when conventional pharmacological treatments, multidisciplinary pain management methods, and/or spinal surgery had not been successful (26). In a broader context, Nadeau and colleagues (15) reported that for patients with chronic cancer pain, physicians in the United States treating chronic pain related to cancer with opioids, appear to be particularly conservative in their use of orally administered opioids, seldom exceeding 300 morphine milligram equivalents daily, and hence a tendency to switch to IT opioid administration even though oral regimens may have sufficed if properly managed (15).

Our study recruited 49 adults, 96% of whom had experienced chronic non-cancer pain for longer than five years. Participants with chronically implanted pumps delivering IT opioids reported significantly lower pain severity, pain interference, anxiety, depression, stress, rumination, magnification, helplessness, and catastrophizing, as well as higher self-efficacy, when compared with approximately 13,000 participants in the ePPOC dataset sample (3) upon entry to 36 pain clinics across Australia and New Zealand. Our study utilised this entry to pain unit data as a surrogate baseline with which to measure change over time with treatment.

The use of ePPOC data from 13,343 patients collected at initial visit to one of 36 pain clinics in Australia and New Zealand as surrogate baseline data for the present study has been useful, but has limitations. The large ePPOC data set does not include details of pain treatments or medication (specifically opioids) prior to initial presentation at a pain clinic. In the period included in the ePPOC data collection (2013–2016), opioid use was widespread in primary practice in Australia. It is likely that all patients in the study group were receiving significant amounts of opioids via systemic routes prior to implantation of the IDDS. Historical baseline data for the patients in the present study would likely have reflected this substantial systemic opioid use.

Our findings suggest that long-term IT administration of opioids for a select group of patients, via a chronically implanted pump, may provide benefit as part of a long-term pain management plan. Our data shown in Figure 1 show considerable inter-patient variability in patient outcomes and this finding may be underpinned by the fact that the two patient populations were at different stages in their pain journeys. Specifically, 96% of our patient group had been in active treatment at the pain facility for longer than 5 years, whereas those that contributed to the Nicholas et al. ePPOC dataset were assessed on their first presentation to a pain clinic (3), although they may have seen multiple different health practitioners prior to being referred to the pain clinic.

In our study, demographic data was similar to previously published groups. A high proportion of participants reported an injury at work as the triggering event for their pain. This is aligned with the findings of Herring et al. (2), where 75% of patients receiving IT opioids for pain relief reported an injury had instigated their pain. Similar to the findings of Schultz et al. (34), a high proportion of current study participants had multiple comorbidities unrelated to their pain condition. Spinal pain back/spine/sacrum was identified as most frequent pain site, concordant with other studies where patients received IT opioids (5, 10, 13, 34–36). Study participants had varying numbers of pain sites consistent with the data reported by Nicholas et al. (3) and data reported by Duarte et al. (35) for patients receiving IT opioids delivered via implanted pumps.

86% of our study participants were taking no or only one oral analgesic, concordant with the findings of three previous studies in patients receiving IT opioids for chronic non-cancer pain management (17, 23, 36). However, research by D'Souza et al. (37) showed opposite findings with an increase in opioid dosage both perioperatively and postoperatively observed in patients with long-term chronic non-cancer pain.

BPI outcomes showed strong relationships between patients receiving IT opioids and decreased pain severity and interference compared with patients on first admission to pain clinics. Participants who qualified for IT opioid pumps can be assumed to have had “severe” pain scores and interference prior to pump insertion. This is consistent with IT opioid delivery devices being reserved as a last resort treatment measure for the most difficult to treat patients with the most recalcitrant pain conditions. Therefore, the lower severity observed in the current study group [2.1 points lower than Nicholas et al.'s sample (3)] indicates that long-term IT opioids, delivered via implanted pumps have contributed to this outcome. These findings are in accordance with Hamza et al. (13) and Duarte et al. (35), who also showed reduced pain severity on BPI scores in patients receiving IT opioids via implanted pumps. Hamza et al. (13) also showed decreased interference comparing baseline data (before pump insertion) to follow-up. Recorded comments by study participants receiving IT opioids via implanted pumps (Supplementary Table S1) on increased work capacity and improved quality of life, are aligned with comments made by participants in other published research (34).

DASS outcomes demonstrate strong correlations between patients receiving IT opioids via implanted pumps, and decreased depression, anxiety and stress consistent with others who showed decreased depression and anxiety in patients with implanted pumps for IT opioid administration (35, 36). However, contrary findings have been reported where no differences in depression, significant stress or mental sum scores were observed when compared with Nicholas et al.'s sample (10). Authors acknowledge observed psychosocial improvements are likely to have been secondary effects due to a reduction in pain, rather than directly attributable to IT drug administration via the IDDS.

Herein, this research is the first to show strong correlations between helplessness and overall catastrophizing, and IT opioid therapy, as well as correlations between rumination and magnification, and IT opioid therapy. Lower catastrophizing scores are associated with positive mental health outcomes, which are vital for individuals coping with chronic pain.

Scores on the PSEQ showed strong correlations between participants with implanted pumps for IT opioid delivery and high self-efficacy, as compared with patients upon first presentation to a pain clinic. Past research has shown that higher self-efficacy is associated with reduced pain (5). Current study results are in accordance with Duarte et al. (35), who found increased self-efficacy in patients receiving IT opioids via implanted pumps when assessed by a multidisciplinary team. Given the importance of self-beliefs on chronic pain outcomes, this finding has importance.

The outcomes of this study provide a comprehensive picture of the chronic pain experience in this special population and the impact of IT opioid therapy on the perception of pain, and supports the benefits of this treatment modality for their ongoing care. However, it is important to note the complications associated with such devices. These include the invasiveness of implantation, the risk of infection, the risk of device failure and the downstream ramifications from these problems (38). Our findings provide an important contribution to the otherwise sparse landscape of literature regarding quality-of-life measures for individuals with chronic non-cancer pain managed by IT opioids delivered via implanted pumps.

The main limitation of this study was the lack of historical baseline data for this legacy patient group, limiting generalizability of the results and correlation of improved outcomes directly with IT opioid therapy. Another limitation of this study was the unbalanced sample sizes of patients with chronically implanted pumps for IT opioid administration (*n* = 49) and the ePPOC dataset (*n* = 13,343) published by Nicholas et al. (3). Social desirability may also have impacted findings, with participants potentially giving positive answers to the self-report questions to please research interviewers. Future larger scale multicenter studies would be valuable. Also, the reliance on legacy patients as participants in the IT opioid pump group, had the potential to introduce selection bias and limit generalizability due to the impact of long-term specialist care on pain outcomes. Patients who had previously been treated with IT opioids without success would have been lost from the current patient cohort of legacy patients contributing a bias towards positive outcomes. Potential confounding variables such as the duration of prior treatments and psychological comorbidities were also not recorded as part of this study and may have impacted outcomes. The impact of polypharmacy on pain outcomes is another consideration as many patients included in the study were also taking supplementary analgesics or other medications for co-morbid conditions.

## 5 Conclusions

This study demonstrated the utility of the large data ePPOC initiative of the Faculty of Pain Medicine of the Australian and New Zealand College of Anaesthetists. Published ePPOC data has provided justifiable surrogate baseline data allowing opportunities for outcomes research into select patient treatment groups where actual historical baseline data does not exist. This study indicates broader biopsychosocial impacts from IT opioid therapy for chronic non-cancer pain management in a select patient population. This work highlighted the difficulties in conducting research with this legacy patient population due to comorbidities and the lack of historical baseline data for these patients.

Compared with the ePPOC study group upon initial presentation to one of 36 pain clinical in Australia and New Zealand, participants in the present study who received long-term IT opioids reported significantly lower (*p* ≤ 0.001) pain severity (4.3 vs. 6.4), and pain interference scores (5.5 vs. 7), significantly lower depression (20.2 vs. 13.7), anxiety (9.6 vs. 14.1), stress (15.5 vs. 21), rumination (6.9 vs. 10), magnification (3.8 vs. 5.9), helplessness (9.7 vs. 14.1), general catastrophizing (20.4 vs. 29.8), and higher self-efficacy (29.5 vs. 20.7).

Our present data may assist clinicians considering use of, or currently managing patients with, implanted IT drug delivery devices. It is important to note that the improved outcomes for patients using long-term IT opioids administered via IDDS relative to the surrogate baseline ePPOC data, did so as part of a comprehensive pain management plan. Furthermore, as the process of IT pump implantation is highly invasive, the IT route of drug administration is only used for pain relief when traditional routes of analgesic/adjuvant drug administration prove inadequate due to poor efficacy and/or dose-limiting adverse effects (6).

The stated limitations of this study preclude definitive conclusions regarding improved efficacy of IT opioids, however, there are still important findings to consider. The study results allow suggestion that long term IT opioids may be associated with superior achievable efficacy and/or less side effects than high dose opioids by conventional routes in patients with chronic non-cancer pain that failed to respond to administration of opioids and other analgesic/adjuvant agents delivered by systemic routes. The increased efficacy and reduced systemic side effects can be considered to have contributed to the demonstrated improved outcomes of pain control, function and psychological well-being.

These conclusions are suggested, but not established by this study, and are instead, a basis to support further specific studies on these topics. Future research should consider a randomized clinical trial or pre-post investigation on IT opioid pump management to obtain a more direct comparison.

## Data Availability

The raw data supporting the conclusions of this article will be made available by the authors, without undue reservation.

## References

[1] Chiarotto A Falla D Polli A Monticone M. Validity and responsiveness of the pain self-efficacy questionnaire in patients with neck pain disorders. J Orthop Sports Phys Ther. (2018) 48(3):204–16. 10.2519/jospt.2018.7605 29257925

[2] Herring EZ Frizon LA Hogue O Mejia JU Rosenquist R Bolash RB Long-term outcomes using intrathecal drug delivery systems in complex regional pain syndrome. Pain Med. (2019) 20(3):515–20. 10.1093/pm/pny104 29889241

[3] Nicholas MK Costa DSJ Blanchard M Tardif H Asghari A Blyth FM. Normative data for common pain measures in chronic pain clinic populations: closing a gap for clinicians and researchers. Pain. (2019) 160(5):1156–65. 10.1097/j.pain.0000000000001496 30694928

[4] Holloway D Allingham S Bryce M Cameron K Cook M Shebeshi D. A decade of outcomes: the evolution of an australasian outcomes collaboration for chronic pain services. Front Pain Res. (2023) 4:1153001. 10.3389/fpain.2023.1153001 PMC10150651 37139341

[5] Hayek SM McEwan MT Veizi E DeLozier SJ Pogrebetskaya M. Effects of bupivacaine on opioid patient-controlled intrathecal analgesia in chronic pain patients implanted with drug delivery systems. Pain Med. (2021) 22(1):22–33. 10.1093/pm/pnaa076 32289829

[6] Chiarotto A Vanti C Ostelo RW Ferrari S Tedesco G Rocca B The pain self-efficacy questionnaire: cross-cultural adaptation into Italian and assessment of its measurement properties. Pain Pract. (2015) 15(8):738–47. 10.1111/papr.12242 25264358

[7] Rezai A Kloth D Hansen H Schultz D Thomson S Deer T Physician response to medtronic’s position on the use of off-label medications in the synchromed pump. Neuromodulation. (2013) 16(5):398–400. 10.1111/ner.12108 24581423

[8] Karri J Singh M Modi DJ Orhurhu V Seale C Saulino M Combination intrathecal drug therapy strategies for pain management. Pain Physician. (2021) 24(8):549–69. 34793643

[9] Smith MT. Neuroexcitatory effects of morphine and hydromorphone: evidence implicating the 3-glucuronide metabolites. Clin Exp Pharmacol Physiol. (2000) 27(7):524–8. 10.1046/j.1440-1681.2000.03290.x 10874511

[10] Sommer B Karageorgos N AlSharif M Stubbe H Hans FJ. Long-term outcome and adverse events of intrathecal opioid therapy for nonmalignant pain syndrome. Pain Pract. (2020) 20(1):8–15. 10.1111/papr.12818 31291509

[11] Sinclair SJ Siefert CJ Slavin-Mulford JM Stein MB Renna M Blais MA. Psychometric evaluation and normative data for the depression, anxiety, and stress scales-21 (DASS-21) in a nonclinical sample of U.S. Adults. Eval Health Prof. (2012) 35(3):259–79. 10.1177/0163278711424282 22008979

[12] Atli A Theodore BR Turk DC Loeser JD. Intrathecal opioid therapy for chronic nonmalignant pain: a retrospective cohort study with 3-year follow-up. Pain Med. (2010) 11(7):1010–6. 10.1111/j.1526-4637.2010.00876.x 20492572

[13] Hamza M Doleys D Wells M Weisbein J Hoff J Martin M Prospective study of 3-year follow-up of low-dose intrathecal opioids in the management of chronic nonmalignant pain. Pain Med. (2012) 13(10):1304–13. 10.1111/j.1526-4637.2012.01451.x 22845187

[14] De Andres J Hayek S Perruchoud C Lawrence MM Reina MA De Andres-Serrano C Intrathecal drug delivery: advances and applications in the management of chronic pain patient. Front Pain Res. (2022) 3:900566. 10.3389/fpain.2022.900566 PMC9246706 35782225

[15] Nadeau SE Wu JK Lawhern RA. Opioids and chronic pain: an analytic review of the clinical evidence. Front Pain Res. (2021) 2:721357. 10.3389/fpain.2021.721357 PMC8915556 35295493

[16] Liu C-S Zheng Y-R Zhang Y-F Long X-Y. Research progress on berberine with a special focus on its oral bioavailability. Fitoterapia. (2016) 109:274–82. 10.1016/j.fitote.2016.02.001 26851175

[17] Kleinmann B Firoozabadi NK Wolter T. A cross-cultural perspective on intrathecal opioid therapy between German and Iranian patients. Cult Med Psychiatry. (2021) 45(2):218–33. 10.1007/s11013-020-09682-6 32725439 PMC8463370

[18] Erdemoglu A Koc R. Brief pain inventory score identifying and discriminating neuropathic and nociceptive pain. Acta Neurol Scand. (2013) 128(5):351–8. 10.1111/ane.12131 23594114

[19] Keller S Bann CM Dodd SL Schein J Mendoza TR Cleeland CS. Validity of the brief pain inventory for use in documenting the outcomes of patients with noncancer pain. Clin J Pain. (2004) 20(5):309–18. 10.1097/00002508-200409000-00005 15322437

[20] Sullivan M. The pain catastrophising scale user manual. (2009).

[21] Statistics Kingdom. Two Sample T-Test Calculator (Pooled-Variance) (2017). Available online at: https://www.statskingdom.com/140MeanT2eq.html (Accessed August 28, 2024).

[22] Tardif H Arnold C Hayes C Eagar K. Establishment of the australasian electronic persistent pain outcomes collaboration. Pain Med. (2017) 18(6):1007–18. 10.1093/pm/pnw201 27524828

[23] Yoo Y Oh JH Lee H Choi H Joo S Han AH Myth and truth in opioid consumption with intrathecal morphine pump implantation in chronic pain: a retrospective cohort study with claims database in South Korea. Pain Med. (2022) 24(1):79–88. 10.1093/pm/pnac110 35881702

[24] University of Wollongong. ePPOC Clinical Reference Manual. Wollongong: University of Wollongong (2021).

[25] Cleeland CS Ryan KM. Pain assessment: global use of the brief pain inventory. Annals, academy of medicine. Singapore. (1994) 23(2):129–38. 8080219

[26] Lovibond SH Lovibond PF. Manual for the depression anxiety stress scales: Psychology Foundation of Australia. (1996).

[27] Zanon C Brenner RE Baptista MN Vogel DL Rubin M Al-Darmaki FR Examining the dimensionality, reliability, and invariance of the depression, anxiety, and stress scale–21 (DASS-21) across eight countries. Assessment. (2021) 28(6):1531–44. 10.1177/1073191119887449 31916468

[28] Crawford JR Henry JD. The depression anxiety stress scales (DASS): normative data and latent structure in a large non-clinical sample. Br J Clin Psychol. (2003) 42(Pt 2):111–31. 10.1348/014466503321903544 12828802

[29] Andreou E Alexopoulos EC Lionis C Varvogli L Gnardellis C Chrousos GP Perceived stress scale: reliability and validity study in Greece. Int J Environ Res Public Health. (2011) 8(8):3287–98. 10.3390/ijerph8083287 21909307 PMC3166743

[30] Fernandes L Storheim K Lochting I Grotle M. Cross-cultural adaptation and validation of the Norwegian pain catastrophizing scale in patients with low back pain. BMC Musculoskelet Disord. (2012) 13(1):111. 10.1186/1471-2474-13-111 22726668 PMC3407790

[31] Nicholas MK. The pain self-efficacy questionnaire: taking pain into account. Eur J Pain. (2007) 11(2):153–63. 10.1016/j.ejpain.2005.12.008 16446108

[32] iCalcu. Chi-Square Calculator (2022). Available online at: https://www.icalcu.com/stat/chisqtest.html (Accessed August 28, 2024).

[33] Zajacova A Grol-Prokopczyk H Zimmer Z. Pain trends among American adults, 2002–2018: patterns, disparities, and correlates. Demography. (2021) 58(2):711–38. 10.1215/00703370-8977691 33834222 PMC8035485

[34] Schultz DM Orhurhu V Khan F Hagedorn JM Abd-Elsayed A. Patient satisfaction following intrathecal targeted drug delivery for benign chronic pain: results of a single-center survey study. Neuromodulation. (2020) 23(7):1009–17. 10.1111/ner.13167 32378289 PMC7687151

[35] Duarte RV Raphael JH Sparkes E Southall JL LeMarchand K Ashford RL. Long-term intrathecal drug administration for chronic nonmalignant pain. J Neurosurg Anesthesiol. (2012) 24(1):63–70. 10.1097/ANA.0b013e31822ff779 21904220

[36] Kleinmann B Wolter T. Intrathecal opioid therapy for non-malignant chronic pain: a long-term perspective. Neuromodulation. (2017) 20(7):719–26. 10.1111/ner.12617 28560830

[37] D’Souza RS Warner MA Olatoye OO Langford BJ Bruns DL Schroeder DR Perioperative opioid consumption and clinical outcomes in surgical patients with a Pre-existing opioid-based intrathecal drug delivery system. Anesth Analg. (2022) 134(1):35–43. 10.1213/ANE.0000000000005662 34260427 PMC8678135

[38] Awaad Y Rizk T Siddiqui I Roosen N McIntosh K Waines GM. Complications of intrathecal baclofen pump: prevention and cure. Int Sch Res Notices. (2012) 2012(1):575168. 10.5402/2012/575168 PMC3323842 22548189

