# Supplementary material for: Evaluation of long-term outcomes with intrathecal opioid treatment: a comparison utilizing data derived from pain clinic populations in Australia and New Zealand
Source: Front Pain Res (Lausanne). 2025 Feb 14;6:1527371. doi: 10.3389/fpain.2025.1527371 (PMC11868084; doi:10.3389/fpain.2025.1527371)
Supplement: Supplementary file 1 [file Table1.docx]

Evaluation of long-term outcomes with intrathecal opioid treatment: A comparison utilizing comparative data derived from pain clinic populations in Australia and New Zealand

Elouise Rose Comber^1^, Jenny Strong^2^, Orla Moore^3^, Asaduzzaman Khan^2^, James O’Callaghan^4^, Benjamin Manion^4^, Brendan Joseph Moore^4,5^^, Maree Therese Smith^5^^

^1^School of Chemistry and Molecular Biosciences, Faculty of Science, The University of Queensland, Brisbane, Queensland, Australia

^2^School of Health and Rehabilitation Sciences, Faculty of Health and Behavioural Sciences, The University of Queensland, Brisbane, Queensland, Australia

^3^School of Medicine, Faculty of Medicine, The University of Queensland, Brisbane, Queensland, Australia

^4^Axxon Pain Medicine, Brisbane, Queensland, Australia

^5^School of Biomedical Sciences, Faculty of Medicine, The University of Queensland, Brisbane, Queensland, Australia

^contributed equally

Supplementary Material

# Supplementary Tables

**Table S1**

**Comments from Study Participants**

- Research on the impacts of IT medications themselves on cognitive function would be worthwhile.
- Pump has been incredible and has allowed me to travel.
- A study on the patient experience of receiving care for chronic pain would be useful.
- Future studies on the individual financial impact of chronic pain would be useful.
- I only feel like I am okay due to the quality and speed of help available (from pain specialists) when it's needed.
- I can now sit down without pain because of my pump (this was not possible before pump insertion).
- The pump allowed me to return to university and as well as reconnect with friends and family.
- I suffered from opioid addiction before pump insertion. The pump changed my life and my family noticed the change too.
- There is significant doctor (GP) hesitancy regarding the pump. GPs need better knowledge of the pumps.
- I wouldn't have given these questionnaire answers before the pump. The pump has made my life 100% better.
- I was bedridden before my pump. The pump allowed me to return to university and finish my degree.
- I went back to work because of the pump. I could barely move before the pump.
- I couldn't walk before the pump due to pain.
- My pump gave me my life back.

| Table S2  Specific IT pump medications for study participants (n=49) | | | |
| --- | --- | --- | --- |
|  |  | n | % |
| IT pump medications | |  |  |
|  | Morphine | 17 | 34.7 |
|  | Morphine + Clonidine | 4 | 8.2 |
|  | Morphine + Bupivacaine | 3 | 6.1 |
|  | Morphine + Ropivacaine | 2 | 4.1 |
|  | Morphine + Clonidine + Ropivacaine | 1 | 2.0 |
|  | Morphine + Clonidine + Bupivacaine | 2 | 4.1 |
|  | Hydromorphone | 1 | 2.0 |
|  | Hydromorphone + Clonidine | 2 | 4.1 |
|  | Hydromorphone + Bupivacaine | 4 | 8.2 |
|  | Hydromorphone + Baclofen | 1 | 2.0 |
|  | Hydromorphone + Ropivacaine | 1 | 2.0 |
|  | Hydromorphone + Clonidine + Bupivacaine | 2 | 4.1 |
|  | Fentanyl | 3 | 6.1 |
|  | Fentanyl + Bupivacaine | 1 | 2.0 |
|  | Fentanyl + Clonidine | 1 | 2.0 |
|  | Fentanyl + Ropivacaine | 1 | 2.0 |
|  | Fentanyl + Bupivacaine + Clonidine | 1 | 2.0 |
|  | Sulfentanil | 1 | 2.0 |
|  | Sulfentanil + Clonidine + Bupivacaine | 1 | 2.0 |

| Table S3  BPI, DASS, PCS and PSEQ outcomes for patients receiving long-term IT opioids in the current study, relative to normative data for Nicholas et al.’s [23] study participants upon entry to 36 pain clinics across Australia and NZ. | | | |
| --- | --- | --- | --- |
|  |  | **Nicholas et al. Normative Data Study [23] Total Sample** | **Current Study Total Sample** |
| BPI severity | |  |  |
|  | Mean (SD) | 6.4 (1.8) | 4.3 (1.7)  *** |
|  | Median (IQR) | 6.5 (2.5) | 4.8 (2.9) |
|  | n | 12,611 | 49 |
| BPI interference | |  |  |
|  | Mean (SD) | 7 (2.1) | 5.5 (2.7)  *** |
|  | Median (IQR) | 7.3 (2.7) | 6 (4.1) |
|  | n | 12,981 | 49 |
| DASS depression | |  |  |
|  | Mean (SD) | 20.2 (12.8) | 13.7 (13.2)  *** |
|  | Median (IQR) | 20 (22) | 12 (26) |
|  | n | 12,473 | 49 |
| DASS anxiety |  |  |  |
|  | Mean (SD) | 14.1 (10.9) | 9.6 (8.1)  ** |
|  | Median (IQR) | 12 (16) | 8 (11) |
|  | n | 12,432 | 49 |
| DASS stress |  |  |  |
|  | Mean (SD) | 21 (11.5) | 15.5 (11.1)  *** |
|  | Median (IQR) | 20 (18) | 14 (18) |
|  | n | 12,408 | 49 |
| PCS rumination | |  |  |
|  | Mean (SD) | 10 (4.9) | 6.9 (5.8)  *** |
|  | Median (IQR) | 11 (8) | 5 (10) |
|  | n | 12,127 | 49 |
| PCS magnification | |  |  |
|  | Mean (SD) | 5.9 (3.6) | 3.8 (3.4)  *** |
|  | Median (IQR) | 6 (6) | 3 (5.5) |
|  | n | 12,110 | 49 |
| PCS helplessness | |  |  |
|  | Mean (SD) | 14.1 (6.5) | 9.7 (7.5)  *** |
|  | Median (IQR) | 15 (10) | 9 (12.5) |
|  | n | 11,928 | 49 |
| PCS total |  |  |  |
|  | Mean (SD) | 29.8 (13.9) | 20.4 (15.6)  *** |
|  | Median (IQR) | 31 (22) | 19 (24.5) |
|  | n | 12,231 | 49 |
| PSEQ total |  |  |  |
|  | Mean (SD) | 20.7 (13.3) | 29.5 (15.5)  *** |
|  | Median (IQR) | 19 (19) | 24 (24) |
|  | n | 12,718 | 49 |

Continuous variables (means and SDs) were analysed using pairwise independent t-tests; * = p<0.05, ** = p<0.01, *** = p<0.001, ns = no significance. BPI: Brief Pain Inventory; DASS: Depression Anxiety Stress Scale; PCS: Pain Catastrophising Scale; PSEQ: Pain Self Efficacy Questionnaire; SD: standard deviation; IQR: interquartile range; n: sample size.
