# Supplementary material for: Evaluation of long-term outcomes with intrathecal opioid treatment: a comparison utilizing data derived from pain clinic populations in Australia and New Zealand
Source: Front Pain Res (Lausanne). 2025 Feb 14;6:1527371. doi: 10.3389/fpain.2025.1527371 (PMC11868084; doi:10.3389/fpain.2025.1527371)
Supplement: Supplementary file 2 [file Datasheet1.pdf]

Evaluation of long-term outcomes with intrathecal opioid treatment: A comparison utilizing comparative data derived from pain clinic populations in Australia and New Zealand

Elouise Rose Comber[1], Jenny Strong[2], Orla Moore[3], Asaduzzaman Khan[2], James O'Callaghan[4], Benjamin Manion[4], Brendan Joseph Moore[4,5^], Maree Therese Smith[5^]

[1]School of Chemistry and Molecular Biosciences, Faculty of Science, The University of Queensland, Brisbane, Queensland, Australia

[2]School of Health and Rehabilitation Sciences, Faculty of Health and Behavioural Sciences, The University of Queensland, Brisbane, Queensland, Australia

[3]School of Medicine, Faculty of Medicine, The University of Queensland, Brisbane, Queensland, Australia

[4]Axxon Pain Medicine, Brisbane, Queensland, Australia

[5]School of Biomedical Sciences, Faculty of Medicine, The University of Queensland, Brisbane, Queensland, Australia

^contributed equally

Supplementary Material

1 Supplementary Tables

Table S1

Comments from Study Participants

- Research on the impacts of IT medications themselves on cognitive function would be worthwhile.
- Pump has been incredible and has allowed me to travel.
- A study on the patient experience of receiving care for chronic pain would be useful.
- Future studies on the individual financial impact of chronic pain would be useful.
- I only feel like I am okay due to the quality and speed of help available (from pain specialists) when it's needed.
- I can now sit down without pain because of my pump (this was not possible before pump insertion).
- The pump allowed me to return to university and as well as reconnect with friends and family.
- I suffered from opioid addiction before pump insertion. The pump changed my life and my family noticed the change too.

- There is significant doctor (GP) hesitancy regarding the pump. GPs need better knowledge of the pumps.
- I wouldn't have given these questionnaire answers before the pump. The pump has made my life 100% better.
- I was bedridden before my pump. The pump allowed me to return to university and finish my degree.
- I went back to work because of the pump. I could barely move before the pump.
- I couldn't walk before the pump due to pain.
- My pump gave me my life back.

Table S2
Specific IT pump medications for study participants (n=49)

	n	%
**IT pump medications**		
Morphine	17	34.7
Morphine + Clonidine	4	8.2
Morphine + Bupivacaine	3	6.1
Morphine + Ropivacaine	2	4.1
Morphine + Clonidine + Ropivacaine	1	2.0
Morphine + Clonidine + Bupivacaine	2	4.1
Hydromorphone	1	2.0
Hydromorphone + Clonidine	2	4.1
Hydromorphone + Bupivacaine	4	8.2
Hydromorphone + Baclofen	1	2.0
Hydromorphone + Ropivacaine	1	2.0
Hydromorphone + Clonidine + Bupivacaine	2	4.1
Fentanyl	3	6.1
Fentanyl + Bupivacaine	1	2.0
Fentanyl + Clonidine	1	2.0
Fentanyl + Ropivacaine	1	2.0
Fentanyl + Bupivacaine + Clonidine	1	2.0
Sulfentanil	1	2.0
Sulfentanil + Clonidine + Bupivacaine	1	2.0

Table S3
BPI, DASS, PCS and PSEQ outcomes for patients receiving long-term IT opioids in the current study, relative to normative data for Nicholas et al.'s [23] study participants upon entry to 36 pain clinics across Australia and NZ.

		Nicholas et al. Normative Data Study [23] Total Sample	Current Study Total Sample
**BPI severity**			
	Mean (SD)	6.4 (1.8)	4.3 (1.7) ***
	Median (IQR)	6.5 (2.5)	4.8 (2.9)
	n	12,611	49
**BPI interference**			
	Mean (SD)	7 (2.1)	5.5 (2.7) ***
	Median (IQR)	7.3 (2.7)	6 (4.1)
	n	12,981	49
**DASS depression**			
	Mean (SD)	20.2 (12.8)	13.7 (13.2) ***
	Median (IQR)	20 (22)	12 (26)
	n	12,473	49
**DASS anxiety**			
	Mean (SD)	14.1 (10.9)	9.6 (8.1) **
	Median (IQR)	12 (16)	8 (11)
	n	12,432	49
**DASS stress**			
	Mean (SD)	21 (11.5)	15.5 (11.1) ***
	Median (IQR)	20 (18)	14 (18)
	n	12,408	49
**PCS rumination**			
	Mean (SD)	10 (4.9)	6.9 (5.8) ***
	Median (IQR)	11 (8)	5 (10)
	n	12,127	49
**PCS magnification**			
	Mean (SD)	5.9 (3.6)	3.8 (3.4) ***
	Median (IQR)	6 (6)	3 (5.5)
	n	12,110	49
**PCS helplessness**			
	Mean (SD)	14.1 (6.5)	9.7 (7.5)

			***
	Median (IQR)	15 (10)	9 (12.5)
	n	11,928	49
**PCS total**			
	Mean (SD)	29.8 (13.9)	20.4 (15.6) ***
	Median (IQR)	31 (22)	19 (24.5)
	n	12,231	49
**PSEQ total**			
	Mean (SD)	20.7 (13.3)	29.5 (15.5) ***
	Median (IQR)	19 (19)	24 (24)
	n	12,718	49

Continuous variables (means and SDs) were analysed using pairwise independent t-tests; * = p<0.05, ** = p<0.01, *** = p<0.001, ns = no significance. BPI: Brief Pain Inventory; DASS: Depression Anxiety Stress Scale; PCS: Pain Catastrophising Scale; PSEQ: Pain Self Efficacy Questionnaire; SD: standard deviation; IQR: interquartile range; n: sample size.
